# Mitochondrial Arrest on the Microtubule Highway—A Feature of Heart Failure and Diabetic Cardiomyopathy?

**DOI:** 10.3389/fcvm.2021.689101

**Published:** 2021-07-02

**Authors:** Sarah Kassab, Zainab Albalawi, Hussam Daghistani, Ashraf Kitmitto

**Affiliations:** Division of Cardiovascular Sciences, Faculty of Biology, Medicine and Health, School of Medical Sciences, Manchester Academic Health Science Centre, The University of Manchester, Manchester, United Kingdom

**Keywords:** diabetic cardiomyopathy, heart failure, Miro1, microtubules, HDAC6, NLRP3, mitochondrial dysfunction, mitochondrial movement

## Abstract

A pathophysiological consequence of both type 1 and 2 diabetes is remodelling of the myocardium leading to the loss of left ventricular pump function and ultimately heart failure (HF). Abnormal cardiac bioenergetics associated with mitochondrial dysfunction occurs in the early stages of HF. Key factors influencing mitochondrial function are the shape, size and organisation of mitochondria within cardiomyocytes, with reports identifying small, fragmented mitochondria in the myocardium of diabetic patients. Cardiac mitochondria are now known to be dynamic organelles (with various functions beyond energy production); however, the mechanisms that underpin their dynamism are complex and links to motility are yet to be fully understood, particularly within the context of HF. This review will consider how the outer mitochondrial membrane protein Miro1 (Rhot1) mediates mitochondrial movement along microtubules *via* crosstalk with kinesin motors and explore the evidence for molecular level changes in the setting of diabetic cardiomyopathy. As HF and diabetes are recognised inflammatory conditions, with reports of enhanced activation of the NLRP3 inflammasome, we will also consider evidence linking microtubule organisation, inflammation and the association to mitochondrial motility. Diabetes is a global pandemic but with limited treatment options for diabetic cardiomyopathy, therefore we also discuss potential therapeutic approaches to target the mitochondrial-microtubule-inflammatory axis.

## Introduction

Diabetic Mellitus (DM) remains a global epidemic, with an estimated 463 million cases worldwide in 2019, and is associated with marked morbidity and mortality rates (1). Diabetes is a major risk factor for heart failure (HF), with a three-fold higher prevalence for developing coronary artery disease, CAD (2, 3). Although, approximately 50 years ago, it was revealed that myocardial remodelling and dysfunction can also occur in diabetic patients in the absence of CAD, a condition termed diabetic cardiomyopathy (DCM) which commonly advances to HF (4, 5). Recently it has emerged that in addition to HF with reduced ejection fraction (HFrEF) roughly half of all HF cases can be classified as HF with preserved ejection fraction (HFpEF), which is less well-understood in comparison. Further, treatments developed to manage HFrEF have limited efficacy in HFpEF patients; highlighting the need for a more detailed understanding of the disease mechanisms. Significantly, over a third of patients with HFpEF have type 2 diabetes (6, 7) indicating that the diabetic insult leads, at least initially, to the development of a distinct myocardial phenotype.

Mitochondrial dysfunction is a hallmark of HF (8) and more recently identified as a feature of HFpEF (9) and DCM (10). While mitochondrial dysfunction is a broad term a recurring feature of HF both in the presence and absence of diabetes, is morphological remodelling of mitochondria, with reports of both swelling and fragmentation [for a review see (11)]. Mitochondrial size, shape and distribution (factors dictating function) are regulated by mechanisms grouped under the umbrella term “mitochondrial dynamics,” which also encompasses mitochondrial turnover, mitophagy and biogenesis, as reviewed by (12). Mitochondrial movement, although less well-studied, particularly in the heart, is driven by the outer mitochondrial membrane protein Miro1 (also termed RHOT1) and is also believed to be important for regulating and maintaining a healthy mitochondrial network (13).

DCM is a chronic low grade inflammatory condition, with inflammation associated with the pathogenesis of HF and linked to the development of mitochondrial dysfunction [as reviewed recently (14)]. Kaludercic and Di Lisa (15) provided an overview of a number of studies indicating that excessive ROS production due to mitochondrial dysfunction is a causative agent of increased expression and activity of the inflammasome, NLRP3. NLRP3 is a multimeric complex formed from NOD-like receptor 3, the apoptosis-associated speck-like protein containing (ASC) adaptor protein, and caspase-1, (16, 17). Although, details are somewhat sparse (and not within the setting of the heart or DCM) an association between proteins mediating mitochondrial dynamics and NLRP3 inflammasome assembly is also emerging (18, 19).

This review will summarise evidence for the emerging role of dysregulated mitochondrial movement in HF/DCM, the intersection with aberrant mitochondrial dynamics and NLRP3 activity focusing on the involvement of Miro1; as well as considering the putative mechanisms involved.

## Mitochondrial Dynamics in Cardiovascular Health and Disease

Previously perceived as “static” organelles within cardiomyocytes, mitochondria are now known to be highly dynamic undergoing restructuring *via* an equilibrium of fission and fusion (12). In brief, fission, mediated by the GTPase dynamin-related protein 1 (Drp1) and receptors Mitochondrial fission 1 protein (Fis1), Mitochondrial fission factor (MFF), and MiD49/51, leads to the division of a single mitochondrion into two, allowing removal of damaged mitochondria from the network. Whereas fusion, is the amalgamation of two mitochondria into one larger mitochondrion orchestrated by the outer mitochondrial membrane (OMM) proteins Mitofusin 1 (Mfn1) and Mitofusin 2 (Mfn2). Fusion of the inner mitochondrial membrane (IMM) next occurs and is regulated by Optic atrophy 1 (Opa1).

The dynamic nature of mitochondria underpins mitochondrial quality control that is, mitochondrial biogenesis (replacement with healthy mitochondria), maintenance and degradation (mitophagy). Over time mitochondria can accumulate damage as a result of multiple mutations in nuclear-encoded mitochondrial genes or oxidative damage *via* ROS formation as a by-product of OXPHOS. In brief, mitophagy, the removal of damaged mitochondria, is regulated by PTEN-induced kinase protein 1 (PINK1) which upon activation (*via* phosphorylation) recruits cytosolic Parkin (20, 21). Parkin selectively phospho-ubiquitinates the OMM proteins (including Mfn1/2) and facilitates the selective binding and extension of autophagosomes around damaged mitochondria, although details of the exact mechanisms involved remain incomplete (22).

Recently another method of “fusion” has been identified in cardiomyocytes *via* the formation of tubular protrusions known as nanotunnels, visualised by live-cell confocal imaging and electron microscopy (23, 24). Nanotunnels serve as a form of direct intercommunication between cardiac mitochondria over micron distances, which allows the exchange of matrix contents between non-adjacent mitochondria. Whether the frequency of nanotunnels is correlated to cell stress is not yet clear, although links to an imbalance in Ca^2+^ cycling has been implicated (25).

There is a plethora of studies, both clinical and preclinical, identifying decreased expression of fusion proteins and increased levels of fission proteins in the context of cardiovascular disease (26). For example, depressed levels of Mfn1/2 are a feature of human and rodent hearts with impaired contractility and mitochondrial dysfunction (27, 28). Ablation of Mfn1 and Mfn2 results in dilated cardiomyopathy, impaired mitochondrial respiration and mitochondrial fragmentation (29). Opa1 is also depressed in HF patients (30). Whereas, increased fission, fragmented mitochondria and cell death associated with Drp1 expression has been reported as a feature of HF (31). Consequently, research has focused upon developing pharmacological inhibitors of Drp1, for example, treatment of HL-1 cardiomyocytes with mitochondrial division inhibitor-1 is shown to be cardioprotective against ischemia/reperfusion injury (32). However, the Janus nature of Drp1-mediated pathways should not be ignored as in some conditions promotion of these pathways can be cardioprotective (33). Similarly, reduced levels of PINK1 and Parkin in the heart are also associated with ventricular hypertrophy, mitochondrial swelling (34) and disorganised mitochondria (35). Andres et al. (36) also reported how simvastatin provides cardioprotection by triggering Parkin-dependent mitophagy. A balance between mitochondrial fusion, fission and mitophagy is crucial for maintaining a healthy population of mitochondria.

## Miro1 Plays a Central Role Governing Mitochondrial Movement

While Mfn1, Mfn2, Opa1 and Drp1 (and receptors) are essential for regulating mitochondrial size and shape, important for mitochondrial “quality control,” the movement of mitochondria within the cell is also a crucial factor with distribution tightly linked to cellular energy requirements. An elegant study from Bers and colleagues not only captured, using live imaging, fusion and fission events within cardiomyocytes but also tracked mitochondrial movement (37). Interestingly, the study showed that over a 1 h period the net movement of mitochondria between the sarcomeres (interfibrillar, IFM) was <0.3 μm compared to those adjacent to the nucleus (peri-nuclear, PNM) which traversed 2.8 μm; this difference in motility may be due to the IFM being more spatially restricted by the sarcomeric organisation.

Miro1 localised to the OMM, has been firmly established in neurons as central for regulating mitochondrial movement in response to temporal and spatial metabolic demands (13), with impaired mitochondrial trafficking associated with several neurodegenerative diseases (38). Miro1 is also highly expressed in the heart (39) and although less well-studied, knock-down of Miro1 in H9c2 cardiomyoblasts revealed a similar effect upon mitochondrial movement in a Ca^2+^ dependent manner (40). Interestingly, studies exploring mitochondrial transfer *via* transplantation of human induced pluripotent mesenchymal stem cells (iPSC-MSCs) for tissue regeneration in models of anthracycline-induced cardiomyopathy identify the intrinsically high Miro1 content of iPSC-MSCs as essential for facilitating mitochondrial relocation and improved cardiac bioenergetics (41). In contrast, a recent study suggested that knockdown of Miro1 in cultured neonatal cardiomyocytes (NRCMs) could be protective against phenylephrine-induced hypertrophy through attenuating mitochondrial fission (42). Whilst different cardiac pathologies likely require different approaches in terms of therapeutic targeting, NRCMs as a model system may not always be directly translatable to the mature cardiomyocyte in which mitochondria show a substrate preference for free fatty acids rather than pyruvate (product of glycolysis); Dorn et al. have proposed that after birth there is cell-wide replacement with “adult” mitochondria (43). It is noteworthy that Miro1 is also decreased in pancreatic cells of patients with type 2 diabetes, with a mouse model of islet Miro1 ablation developing insulin resistance, increased production of ROS, inflammation and dysregulated mitophagy (44). Evidence, mainly from studies of neuronal tissue (38), indicates that loss of Miro1 is a decisive factor leading to “arrested” motility and linked to the accumulation of damaged mitochondria.

## A Miro1-Macromolecular Complex Mobilises Mitochondria Along Microtubules

**Figure 1:**
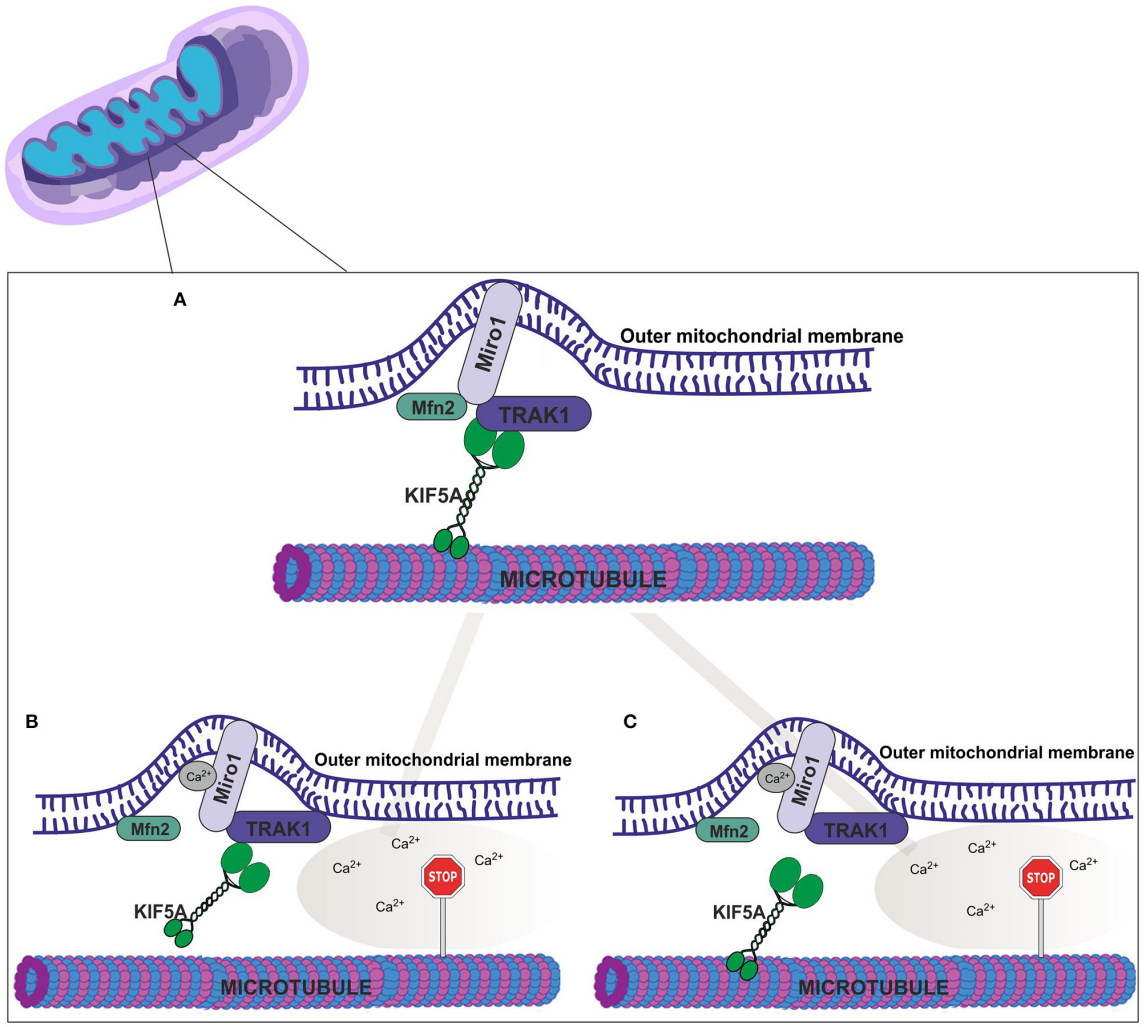
Putative molecular mechanisms of mitochondrial movement. **(A)** In the absence of cytosolic Ca^2+^ (nM) Miro1 (bound to the outer mitochondrial membrane) coordinates mitochondrial movement along microtubules *via* KIF5A, TRAK1 and Mfn2. According to literature Ca^2+^ binding leads to two possible scenarios; **(B)** Conformational changes to Miro1, and subsequent detachment from Mfn2 and release of KIF5A from the microtubule trafficking apparatus, or **(C)** Detachment of Miro1 from Mfn2 and a disruption between TRAK1 and KIF5A interactions. Both scenarios result in halted mitochondrial movement.

Miro proteins bind to kinesin-1/KIF5 and Milton (also known as trafficking kinesin-binding protein 1, TRAK1 or OIP106) (39); interactions proposed to link mitochondria to the microtubule trafficking apparatus (45, 46). Miro1 also directly interacts with Mfn2 in neuronal cells, an association that is proposed as an essential step mediating mitochondrial movement (47) as shown in Figure 1; although, the molecular basis of this interaction remains unknown as is whether this association occurs in cardiomyocytes. Significantly, as discussed above, cardiac Mfn2 levels are reported to be down-regulated in models of DCM (48). How the loss of Mfn2 (which presumably leads to reduced Miro1-Mfn2 interactions) impacts mitochondrial movement and DCM linked phenotypic changes has yet to be clarified.

Ca^2+^ binding to Miro1, *via* two EF-hands within the primary sequence, elicits a conformational change, mediating the association and dissociation of the Miro1-complex assembly from the microtubules, MTs, (with detachment halting mitochondrial motility). This process underpins mitochondrial moment (Figure 1) (49). Aberrant Ca^2+^ homeostasis is a hallmark of HF in patients (50) and associated with DCM (51). Mitochondria play a central role in regulating cytosolic [Ca^2+^] and maintaining the cellular redox status (52). Organisation of mitochondria straddling either side of the dyad (formed by t-tubules, specialised regions of the sarcolemma, and junctional sarcoplasmic reticulum) is essential for Ca^2+^ uptake into the mitochondria for driving bioenergetics (52, 53). While HF is associated with displacement of mitochondria (54), how impaired Ca^2+^ cycling influences mitochondrial movement along microtubules has yet to be examined in detail. Importantly, the Bers group have shown in isolated cardiomyocytes that under stress conditions, causing mitochondrial damage, there is migration of IFM (fission products) *via* MTs to the perinuclear region where they undergo mitophagy (37).

## Microtubule Organisation and Cardiovascular Function and Disease

The presence of MTs in cardiomyocytes has been known for many years (55). MTs are formed by the polymerisation of α/β-tubulin dimers assembling into rods roughly ~ 25 nm in diameter which can extend up to tens of microns in length (56). Similar to mitochondria, MTs are organised into spatially distinct populations; (i) interfibrillar (55), (ii) those surrounding the nuclear envelope (57), and (iii) cortical MTs perpendicular to myofibrils (58). These differing populations of MTs are commonly believed to explain, in part, the diverse physiological roles of MTs in cardiomyocytes, for example, ion channel trafficking, mechanical signalling pathways fundamental for cardiac contractility and inter-organelle communication, as reviewed by Caporizzo et al. (59).

As also discussed in (59) there are numerous studies linking changes to MT properties to the development and progression of cardiovascular diseases. MT remodelling has been identified as the source of increased mechanical stiffness occurring in the early stages of diastolic dysfunction. Accordingly, there has been interest in the use of reagents that depolymerise MTs. Specifically, post-translational modification (PTM) (detyrosination) of MTs is associated with increased viscoelastic resistance in human failing hearts (60). Stiffening of the myocardium due to MT remodelling in the failing heart is also reported to displace mitochondria adjacent to the sarcolemma (subsarcolemmal mitochondria, SSM) triggering the propagation of abnormal Ca^2+^ transients, leading to arrhythmogenesis (8). However, since mitochondria have been demonstrated to provide a bridge between calcium cycling (and contractility) and mechano-electric and chemical MT-mediated inter-organelle tethering (61) the impact of disrupting the MT network upon mitochondrial motility and function needs further investigation.

## Protein Post-Translational Modifications (PTMs) and Role in Mitochondrial Motility

In addition to Ca^2+^ homeostasis, regulatory mechanisms mediating mitochondrial motility include the cellular redox balance (62), as well PTMs of mitochondrial (63) and MT proteins (64). Miro1 is also regulated by PTMs being ubiquitinated after phosphorylation *via* PINK1, triggering Parkin and proteasomal pathways leading to Miro1 degradation (65). Given that the PINK1-Parkin pathway is activated in response to depolarisation of the mitochondrial membrane it is generally considered, in this context, that prevention of mitochondrial movement through Miro1 phosphorylation-degradation is important for preserving mitochondrial quality by segregating those damaged mitochondria for removal from the cell (66). How removal of damaged mitochondria is linked to the transport of mitochondria *via* Miro1 is not clear, particularly within cardiomyocytes; although the Bers study (37) would suggest that mitochondrial transport of IFM precedes mitophagy. The relationship between mitophagy and Miro1 also appears to be cell-type dependent with Miro1 loss-of-function mutations preventing the induction of mitophagy in neurons (67) but activating mitophagy in fibroblasts (68). Miro1 is also a substrate of the class II histone deacetylase, HDAC6, with acetylation of K105 (murine and rat) corresponding to K92 in human Miro1, involving different lysine residues than those targeted for ubiquitination, indicative of two different processes (69). HDAC6 co-immunopreciptates with Miro1 supporting a direct interaction between the two proteins. Importantly, Kalinski et al. (69) also demonstrated that deactetylation of Miro1 leads to stalled mitochondrial movement with detrimental effects upon axon growth, with pharmacological inhibition or deletion of HDAC6 protecting mitochondria against damage and abnormal mitochondrial clustering.

Notably, one of the main components of MTs, α-tubulin, is also an HDAC6 substrate. Acetylation is important for MT stability, as well-inducing conformationally directed MT organisation for the recruitment of kinesin motor proteins (70). Inhibition of HDAC6 in hippocampal neurons is shown to lead to higher levels of α-tubulin and enhanced mitochondrial motility (71). While the role of MT acetylation remains to be fully understood it is intriguing that HDAC6 separately regulates both Miro1 and MT engagement and disengagement and consequently mitochondrial motility.

Significantly, inhibition of HDAC6 activity is also reported as cardioprotective (72) preventing the development of hypertrophy and fibrosis (73). Further, in the context of DCM, HDAC6 inhibition using tubastatin A (TBA) is shown to be beneficial in a rat model of type 1 diabetes for improving outcomes from ischaemia/reperfusion (I/R) injury (74). Moreover, recently, pan-inhibitors of HDACs have been proposed as novel treatments for treating HFpEF, preserving cardiac function in a small animal model (rat) of hypertension induced LV-dysfunction (75) and a larger animal (feline) model of pressure overload (76).

Increased HDAC6 activity in the failing heart has been known for the past decade (77) but a key question that remains to be fully answered is how is HDAC6 activated? Chen and colleagues demonstrated, using an atrial cell line (HL-1), that mitochondrial dysfunction (impaired OXPHOS) induced by treatment with TNF-α, could be rescued using an inhibitor of HDAC6 (78) indicating a mechanistic link between inflammation, mitochondrial function and HDAC6 activity. Further, HDAC6 inhibition in SH-SY5Y cells (a model for Parkinson's disease) is reported to lead to reduced activation of the inflammasome, NLRP3, inflammatory response concomitant with attenuation of dopaminergic neuronal degeneration (79); although the pathways involved were not described.

## Microtubules Play a Critical Role for NLRP3 Activation

Activation of NLRP3 modulates the release of inflammatory cytokines, IL-1β and IL-18, cell death and fibrosis associated with the pathogenesis of DCM (80). The acetylation of α-tubulin, whilst linked to mitochondrial transport, is also shown to play a role in the movement of NLRP3 inflammasome components along microtubules leading to the subsequent apposition of NLRP3 to mitochondrion-associated ASC (81). Multiple studies have established the link between microtubule dynamics and NLRP3 inflammasome activity, for example (82). Significantly NLRP3 activation is a feature of several cardiac pathologies as reviewed in (83). For example, colchicine (a microtubule polymerisation inhibitor) disrupts microtubule/tubulin dynamics suppressing the activation of the NLRP3 inflammasome. Animal models of myocardial infarction treated with colchicine have improved cardiac performance, improved survival rates and attenuated HF development and inflammatory response (84).

Since the acetylation of α-tubulin/NLRP3 is under the control of HDAC6 it is perhaps not surprising that beneficial effects of using HDAC6 inhibitors in preventing IL-1β generation have been demonstrated (85). Although, one macrophage study concluded that HDAC6 is a negative regulator of NLRP3, due to a direct interaction mediated by the ubiquitin binding domains (86); with this mechanism suggested to mediate NLRP3 transport into aggresomes *via* the microtubule network. More recently, Magupalli et al. also demonstrated in macrophages that specific regions of MTs, the microtubule-organising centre (the centrosome), are the sites for NLRP3 assembly and HDAC6 knockout (and loss of ubiquitin-binding) leads to impaired inflammasome assembly and activation (87). Clearly, there is a complex association between NLRP3 assembly, activation and MTs, which may also be tissue/cell type specific.

An indirect link between Miro1 and NLRP3 has also been identified in a rat pancreatic cells using high-fat and high glucose stressors to mimic T2DM conditions (88). Specifically, cells exhibited dysregulated Ca^2+^ homeostasis, which was suggested to lead to the dissociation of Miro1 from mitochondria and subsequent impaired mitochondrial movement, stalled mitophagy and accumulation of damaged ROS producing mitochondria that in turn triggered activation of NLRP3.

## Concluding Remarks

Here we have highlighted evidence for the physio-pathological role of Miro1-mediated movement of mitochondria along MTs and while the majority of data is from the study of neurons evidence is emerging to support a similar role for Miro1 in the heart (40). Therefore, in addition to studies focussing upon strategies to prevent fission (89, 90) an emerging area for future studies is the delineation of the mechanisms surrounding mitophagy, mitochondrial movement, and role of the Miro1-macromolecular complex. For example, it remains unclear as to whether Miro1 expression and activity influences the processes of fission and fusion and is essential for mitophagy in the heart.

**Figure 2:**
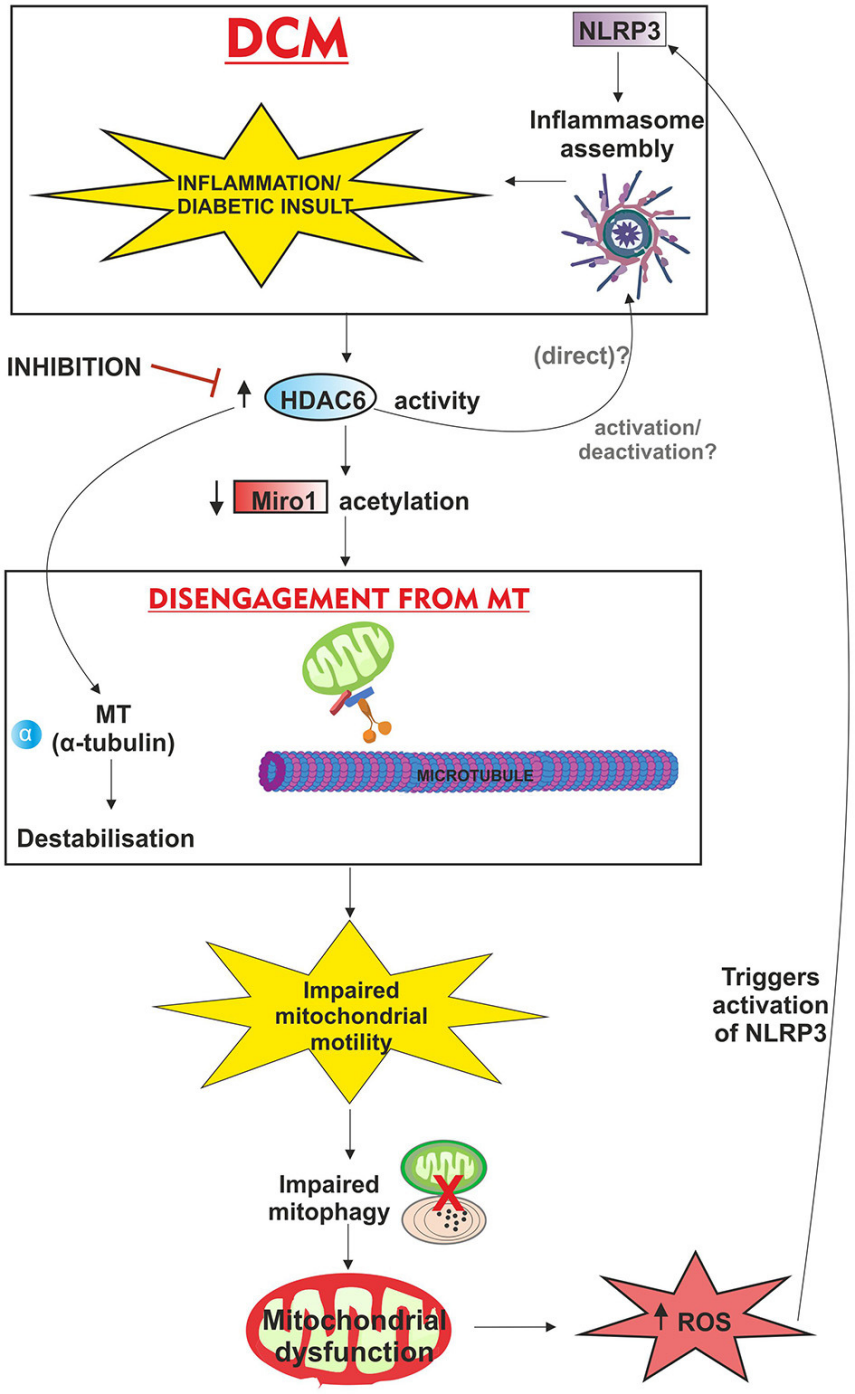
Schematic overview of proposed mechanism of action of HDAC6 and associated deacetylation; linking mitochondrial motility, inflammation and the putative involvement of post-translational modifications in DCM. Inflammation/diabetic insult triggers HDAC6 activity which directly interacts with NLRP3 *via* ubiquitin binding domains (although it is unknown whether this activates/inactivates NLRP3 and inflammasome assembly in the heart). The combination of microtubule (alpha-tubulin) destabilisation and reduced acetylation (important for microtubule stability), contributes to disengagement of the mitochondria from the microtubule apparatus and halts mitochondrial movement. With mitochondrial motility linked to mitophagy, the removal of damaged mitochondria (potentially ROS producing) is impaired, leading to subsequent mitochondrial dysfunction, and oxidative stress further exacerbating inflammation *via* NLRP3. Inhibition of HDAC6 activity is also reported as cardioprotective.

Additionally, we have highlighted a potential link between mitochondrial motility and inflammation and the involvement of deacetylation and HDAC6 (Figure 2). Although technically there remain significant challenges around studying PTMs (91) a better understanding of the functional effects of PTMs on the proteins underpinning mitochondrial motility will provide new avenues for future research. In conclusion, this review article summarises some of the current evidence, and areas where knowledge is lacking, for mitochondrial motility in the heart, and suggests a possible unifying mechanism linking impaired mitophagy, the MT network and the inflammatory response to arrested mitochondrial movement. As the causative agents and mechanisms of mitochondrial dysfunction and impaired motility are discovered, then new promising treatment therapies may emerge for promoting better cardiac outcomes in DCM/HF.

## Author Contributions

AK and SK wrote and planned the manuscript. ZA and HD contributed to the writing process. SK generated the figures in consultation with AK. All authors contributed and approved the submitted article.

## Conflict of Interest

The authors declare that the research was conducted in the absence of any commercial or financial relationships that could be construed as a potential conflict of interest.
